# Correlation between bone marrow dose volumes and acute hematological toxicity in postoperative gynecological cancer patients

**DOI:** 10.12669/pjms.326.11489

**Published:** 2016

**Authors:** Qian Li, Ming-Hua Jiang, Jing Chen, Wei Liu, Bi-Qing Zhu, E-Mei Lu

**Affiliations:** 1 Qian Li, Department of Radiation Oncology, The Affiliated Cancer Hospital of Nanjing Medical University, Jiangsu Cancer Hospital, No.42, Baiziting, Xuanwu District, Nanjing, 210009, China; 2 Ming-Hua Jiang, Department of Radiation Oncology, The Affiliated Cancer Hospital of Nanjing Medical University, Jiangsu Cancer Hospital, No.42, Baiziting, Xuanwu District, Nanjing, 210009, China; 3 Jing Chen, Department of Radiation Oncology, The Affiliated Cancer Hospital of Nanjing Medical University, Jiangsu Cancer Hospital, No.42, Baiziting, Xuanwu District, Nanjing, 210009, China; 4 Wei Liu, Department of Radiation Oncology, The Affiliated Cancer Hospital of Nanjing Medical University, Jiangsu Cancer Hospital, No.42, Baiziting, Xuanwu District, Nanjing, 210009, China; 5 Bi-Qing Zhu, Department of Radiation Oncology, The Affiliated Cancer Hospital of Nanjing Medical University, Jiangsu Cancer Hospital, No.42, Baiziting, Xuanwu District, Nanjing, 210009, China; 6 E-Mei Lu, Department of Radiation Oncology, The Affiliated Cancer Hospital of Nanjing Medical University, Jiangsu Cancer Hospital, No.42, Baiziting, Xuanwu District, Nanjing, 210009, China

**Keywords:** Chemotherapy, Intensity-modulated radiotherapy, Principal component analysis, Whole pelvic radiotherapy

## Abstract

**Objective:**

To identify the association between radiation dose volume and acute hematological toxicity (HT) in postoperative gynecological cancer patients receiving whole pelvic radiotherapy (RT) or intensity-modulated RT (IMRT), a principal component regression model was used to calculate HT.

**Methods:**

Women (n=100) receiving with or without chemotherapy RT were retrospectively analyzed, 52 of whom received chemotherapy (paclitaxel and nedaplatin). The pelvis and lumbar vertebrae, defined as the prolong-pelvic bone marrow, were divided into the (1) combined ilium, ischium and pubis and the (2) lumbar vertebrae and the sacrum. The V5-V40 of subsides was calculated. The complete blood counts were recorded weekly. The principal component analysis was performed on volumes which generated the principal components (PCs), followed by using a logistic regression model.

**Results:**

Forty-seven patients presented with grade 2-3 HT during RT. Chemotherapy increased the incidence of HT compared with RT alone (70.21% vs. 29.79%; p=0.001). Fifty-three patients with persistent HT developed more serious HT at an earlier stage of RT. The chemotherapy cycles and three PCs associated with grade 2-3 HT was identified to form the resulting principal logistic regression model.

**Conclusion:**

A new method to calculate the NTCP was achieved by PCs logistic regression.

## INTRODUCTION

Hematological toxicity (HT) is a common side effect during the course of cancer treatments, reaching up to 60%.^1^,^2^ Chemoradiotherapy is the standard treatment for most locally advanced gynecological cancer patients,^3^,^4^ but this has been observed to increase the risk of HT, which may prolong hospitalization and increase the requirements for transfusions and growth factors.

Approximately 60% of the body’s total bone marrow (BM) is located in the pelvis and vertebrae, and it is usually included within the pelvic radiotherapy (RT) fields. The alleviation of HT theoretically depends on reducing the BM irradiation areas. BM stem cells are radiosensitive and predisposed to apoptosis damage in RT, resulting in myelosuppression.^5^

Intensity-modulated RT (IMRT) is designed to conform the rigid dose coverage to the planning target volume (PTV) by sparing the surrounding normal tissues.^6^,^7^ IMRT can reduce the volume of irradiated BM, especially with lower doses.^8^,^9^ However, it is more difficult to constrain the large extent of the irradiated BM because the pelvic bone and vertebrae are close to the PTV during IMRT, and these bones are contained in the fields during conventional whole pelvic RT.

Normal tissue complication probabilities (NTCP) models derived from dose volume histograms (DVHs) optimize the treatment planning according to biological cost functions, e.g., n, m, and D_50_ for the Lyman-Kutcher-Burman model^10^-^12^ or s, γ and D_50_ for the seriality model.^13^ However, the collinearity among the dose volume parameters confounds the complication probabilities, resulting in inaccurate statistical results. We analyzed the collinearity and summarized the principal components (PCs) of the dose array to a logistic regression. We used a PCs logistic regression model rather than the original dose volume model to formulate a new method of calculating NTCP.

## METHODS

One hundred patients with gynecologic malignancies who received conventional whole pelvic RT (AP/PA fields) or IMRT after transabdominal surgery between January 2012 and June 2013 were analyzed. All of the patients initially underwent pelvic computed tomography (CT). Complete blood counts were obtained on a weekly basis. Patients were excluded if they had received either extended-field (paraaortic) RT or four-field box technique treatments. The study design was approved by the appropriate ethics review boards, and written consent has been obtained from all patients.

### Chemotherapy administration

The concurrent and previous history of chemotherapy was recorded. Some patients (6 in Group one and two in Group II) had both concurrent and prior chemotherapy; thus, these patients were repeatedly calculated in the chemotherapy subgroups. The concurrent chemotherapy was delivered every three weeks (180 mg of paclitaxel on day one, and 100 mg or 30 mg of nedaplatin on day one or on days one, two and three). The patients who received chemotherapy before RT had a higher dosage of paclitaxel and nedaplatin (180-240 mg of paclitaxel and 100-120 mg of nedaplatin). The patients received one (21%), two (17%), three (4%), four (5%), five (1%) or more than six (4%) cycles of chemotherapy.

### Radiation planning

Group I consisted of the patients receiving conventional whole pelvic RT, and Group II consisted of the patients receiving IMRT. The radiation dose was 40-50 Gy given through five treatments per week and 1.8-2.0 Gy per daily fraction. The patients’ characteristics are listed in Table-I.

**Table-I:** Patient characteristics.

	Number of patients	%	Mean	SD
Age			48	10.34262
Group I: Conventional RT	77	77		
Group II: IMRT	23	23		
Weight (kg)			59.19	8.305
Dose fraction (Gy)			1.8773	0.07536
*Prior chemotherapy*				
No	53	53		
Yes	47	47		
*Concurrent chemotherapy*				
No	85	85		
Yes	15	15		
*Chemotherapy (total)*				
No	48	48		
Yes	52	52		
Block				
No	45	45		
Yes	55	55		
*Histological feature*				
Squamous carcinoma	64	64		
Adenocarcinoma	29	29		
Others	7	7		
*Pelvic LN metastasis*				
Negative	65	65		
Positive	17	17		
No scavenge or surgery records lost	18	18		
Days between surgery and radiation			173.47	570.9225
*Diabetes*				
No	97	97		
Yes	3	3		
*Hypertension*				
No	88	88		
Yes	12	12		
Transfusion (during surgery)	25	25		

RT:radiation therapy; IMRT:intensity-modulated radiation therapy; LN:lymph nodes.

Seventy-seven of the patients in Group I received a conventional whole pelvic RT, extending from the bottom of the obturator foramina to the L4-5 interspace or above L4. The lateral border was 1.5 cm lateral to the widest diameter of the pelvic inlet. Blocks were used in 55 of the patients’ treatment plans.

Twenty-three patients received IMRT within co-planar seven- or nine-field plans using 6-MV photons that were generated by commercial inverse treatment planning (Varian Eclipse platform 10.0). The clinical target volume (CTV) was contoured following the previous consensus on the recommendation for all patients, including the upper segment of the vagina, parametrial tissues and lymph node regions (common, external, internal iliac and presacral lymph node regions),^14^ and a 7-mm margin around the vessels was maintained, excluding bone or muscle.

The pelvis and lumbar vertebrae were deemed to be prolong-pelvic bone marrow (P-PBM), which was divided into two, subsides:

The combined ilium, ischium and pubis (IL extending from the iliac crests to the inferior border of the ischial tuberosities, including the acetabula and pubes)

The lumbar vertebrae and sacrum (LA) extending from the superior border of L1 to the entire sacrum. Dose-volume histograms (DVHs) were calculated for each patient from 5 Gy to 40 Gy at a 5-Gy dose-level interval (20 patients did not receive CT examinations in our center; thus only the absolute volume parameters were recorded). These dosimetric parameters were noted as follows:

From IL-V5 to IL-V40
From LA-V5 to LA-V40

### Hematological toxicity

HT was graded according to the Radiation Therapy Oncology Group’s acute radiation toxicity scoring criteria.^15^ The endpoints of interest were the white blood count (WBC), absolute neutrophil count (ANC), hemoglobin (Hgb), and platelet count nadirs, and the highest grade for each of these endpoints was recorded 120 days after the start of the RT. An HT grade of 2-3 was noted as an event. None underwent grade 4 HT. Transfusion was recorded in twenty-five patients in Group I during surgeries, while no patient in Group II had transfusion or none of the patients did during RT.

A proportion of the patients had HT before RT due to prior chemotherapy, and these patients received treatments including granulocyte-monocyte colony stimulating factor (CSF), erythropoietin (EPO) and interleukin-11 (IL-11) before RT to ensure normal blood cell levels. We recorded the first appearance of HT and the start of RT; thus, the interval time in these patients was negative. We also observed that some of the patients had persistent HT during RT; therefore, we defined persistent HT as weekly complete blood counts below the normal value ≥3 times.

### Statistical analysis

All of the DVHs original parameters were transformed to standardized values by the Z-score formula. The standardized values were used to construct a new dataset.

A principal component analysis (PCA) was performed on this new data array (N×N, where N is the number of DVHs parameters) by a correlation matrix. λ_1_≥λ_2_≥…≥λ_i_ denote the eigenvalues of PC i (i=1, 2, 3…N), and e_i_ denotes the eigenvector associated with eigenvalue λ_i_ which is calculated as follows:

e_i_ = C_i_/√λ_*i*_, where C_i_ is the it PC factor loading.

A stepwise logistic regression approach was adopted to identify all PCs with higher grade HT for further analyses. The patients’ factors (age, weight, chemotherapy, dose fraction, block, blood transfusion) were also analyzed using regression models. The principal component score function is expressed as follows:

F_i_= e_1i_ zx_1_+ e_2i_ zx_2_+ e_3i_ zx_3_+…+ e_ni_ zx_n_ (zx_n_= standardized values of original data; n=1, 2, 3…N)

The univariate analysis of HT was calculated by the independent t test or chi-square/Fisher exact test. The collinearity was assessed by the tolerance and the variance inflation factor (VIF). A tolerance value of ≤1 or a VIF value of ≥10 demonstrated that the collinearity correlation existed. The statistics was obtained using SPSS 11.0. A p value of <0.05 was considered to be statistically significant. The P-values were obtained from a two-sided test.

## RESULTS

### Patient characteristics

Seventy-seven patients were treated with conventional RT, and 23 patients were treated with IMRT. In all, 47 patients had received prior chemotherapy, and 15 patients received concurrent chemotherapy. Table-II summarizes the dose volumes of the BM in the two groups. Significant differences were achieved from V5 to V15 and from V25 to V40 of the IL (p<0.01) and from V20 to V40 of the LA (p<0.05).

**Table-II:** Comparison of characteristics between Groups I and II.

	Group I (77 patients)	Group II (23 patients)	P
Age	49.68±10.02	42.39±9.57	0.003
Weight (kg)	60.61±8.42	54.5±5.97	0.002
Dose fraction (Gy)	1.85±0.04	1.98±0.07	0.000
Pelvic LN metastasis	21.70%	18.20%	1.000
Prior chemotherapy	44.20%	56.50%	0.297
Concurrent chemotherapy	14.30%	17.40%	0.743
Total chemotherapy	49.40%	60.90%	0.332
Block	71.40%	0.00%	
Hypertension	16.00%	0.00%	0.063
Diabetes	3.90%	0.00%	1.000
Transfusion (during surgery)	32.47%	0.00%	
V5IL	757.55±97.56	875.92±104.96	.000
V10IL	724.22±95.09	828.89±96.15	.000
V15IL	704.3±93.57	777.84±91.96	.003
V20IL	687.33±92.69	674.39±83.1	.575
V25IL	671.77±91.86	554.8±79.71	.000
V30IL	656.01±91.19	423.21±71.92	.000
V35IL	638.43±90.74	299.52±65.86	.000
V40IL	611±90.29	195±55.03	.000
V5LA	342.99±55.6	329.76±42.85	.494
V10LA	335.63±55.06	318.35±41.7	.296
V15LA	331.06±54.45	309.8±41.28	.170
V20LA	327.24±53.99	295.2±40.72	.021
V25LA	323.98±53.68	262.71±44.92	.000
V30LA	320.8±53.46	213.92±46.7	.000
V35LA	317.37±53.41	156.91±39.66	.000
V40LA	312.45±53.28	106.95±32.32	.000

LN:lymph nodes; V5-V40:dose-volume histogram (DVH) calculated from 5 to 40 Gy at 5-Gy intervals; IL:combination of ilium, ischium and pubis; LA:lumbar vertebrae and sacrum. The data are means ± standard deviation.

### Hematological toxicity

Overall, 47 patients (30 from Group I and 17 from Group II) experienced grade 2-3 HT during RT. Four patients ended the treatment due to the unendurable toxicity symptom of pain due to hemorrhoids. An increased risk of a higher grade acute HT was observed in the patients with total chemotherapy compared with RT alone (70.21% vs. 29.79%; p=0.001). Similarly, patients treated with prior chemotherapy experienced more grade 2-3 HT (63.83% vs. 36.17%; p=0.01, Table-III). However, patients in the concurrent chemotherapy subgroup had less of a risk of suffering HT in both of the groups (23.40% vs. 76.60%; p=0.027). Collectively, chemotherapy delivered before RT had a more marked impact on HT than concurrent chemotherapy.

**Table-III:** Univariate analysis of grade 2-3 acute hematological toxicity in the groups.

	Group I		Group II		All	

	No. of patients (Count/%)	P	No. of patients (Count/%)	P	No. of patients (Count/%)	P
Prior chemotherapy		0.001		1		0.001
No	10 (33.33%)		7 (41.18%)		17 (36.17%)	
Yes	20 (66.67%)		10 (58.82%)		30 (63.83%)	
Concurrent chemotherapy		0.013		1		0.027
No	22 (73.33%)		14 (82.35%)		36 (76.60%)	
Yes	8 (26.67%)		3 (17.65%)		11 (23.40%)	
Chemotherapy (total)		0.001		0.643		0.001
No	8 (26.67%)		6 (35.29%)		14 (29.79%)	
Yes	22 (73.33%)		11 (64.71%)		33 (70.21%)	
Prior chemotherapy without concurrent chemotherapy	14 (46.67%)		8 (47.06%)		22 (46.81%)	
Both prior chemotherapy and concurrent chemotherapy	6(20.00%)		2 (11.76%)		8 (17.02%)	
Concurrent chemotherapy without prior chemotherapy	2 (6.67%)		1 (5.88%)		3 (6.38%)	

The patients received chemotherapy before and/or during RT.

Medical treatments (CSF, EPO or IL-11) were provided to the patients with myelosuppression. However, a proportion of these patients still had abnormal blood counts in their next weekly examinations. A total of 53 patients encountered persistent HT (36 patients in Group I) and required more attention during their daily medical treatments. As shown in Table-IV, the patients with persistent HT developed more serious BM complications and were particularly prone to blood count reductions in the early stage of RT (for all: 69.81% vs. 21.27%; 2.68±10.13 days vs.14.85±12.5 days; p=0.000).

**Table-IV:** Univariate analysis of persistent hematological toxicity and interval days.

	Group I		Group II		All	

	Grade 2-3 (count/%)	Interval days (mean ± SD)	Grade 2-3 (count/%)	Interval days (mean ± SD)	Grade 2-3 (count/%)	Interval days (mean ± SD)
Non-persistent hematological toxicity	8(19.51%)	15.63±12.54	2(33.33%)	10.33±12.36	10(21.27%)	14.85±12.5
Persistent hematological toxicity	22(61.11%)	5.17±7.82	15(88.24%)	-2.59±12.5	37(69.81%)	2.68±10.13
P	0.000	0.000	0.021	0.024	0.000	0.000

Interval days:The interval between the date of the first appearance of hematological toxicity and the start of radiation.

### Principal component analysis (PCA) of the dose- volume array

The volumes irradiated from 5 to 40 Gy are associated with myelosuppression and are therefore correlated with each other. We analyzed the collinearity among the different volume levels. The mathematical results showed that collinearity existed in all data levels (from V5_IL_ to V40_IL_ and from V5_LA_ to V40_LA_), and the minimum VIF value was 294.019 of V20_IL_, whereas all tolerance values decreased to values less than 0.1 (the maximum value was 0.03).

**Fig. 1:**
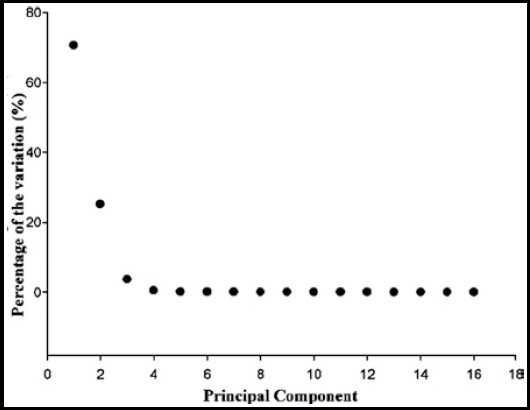
Screen plot displaying the percentage of the variation of each PC in the data array.

We aligned all DVHs parameters into a volume array for each patient. PCs were calculated from the standardized DVH parameters using PCA. Sixteen eigenvectors corresponding to the non-zero eigenvalues of the sample correlation matrix were generated by PCA. Fig.1 shows a screen plot displaying the percentage of the variation in the data array carried by each PC.

### Principal component regression (PCR)

PCR used all PCs of the predictor variables as regressors. Three PCs (1st, 2nd and 16th) and chemotherapy cycles were significantly associated with grade 2-3 HT, as identified in the logistic regression (Table-V).

**Table-V:** Principal component regression results.

Principal component	β value	e value	95%CI	P value
Constant	-1.268			.004
1	-.255	11.295	0.64,0.94	.011
2	.457	4.015	1.13,2.2	.007
16	158.632	5.237E-05	7.80E+24,7.83E+112	.002

CI:confidence interval; e:eigenvalue

To predict a new patient’s toxicity outcome, we applied the coefficient estimated from the regression model to the volume vector such that the principal logistic regression resulting model was expressed as logit P=-1.268+1.082×chemotherapy cycles-0.255×F1+0.457×F2+158.632×F16

PCs were returned to the original data by the Z-score formula and Function (2), resulting in the following final model:

Logit P=1.237 +1.082×chemotherapy cycles-0.071×V5_IL_+0.08×V10_IL_-0.027×V15_IL_+0.014×V20_IL_+0.153×V25_IL_-0.412×V30_IL_+0.454×V35_IL_-0.198×V40_IL_+0.314×V5_LA_-0.541×V10_LA_+0.673×V15_LA_-0.523×V20_LA_0.182×V25_LA_+0.745×V30_LA_-0.91×V35_LA_+0.423×V40_LA_

## DISCUSSION

hematological toxicity (HT) rarely occurs in patients undergoing RT alone. Hematopoiesis injury is rapidly repaired because of the marrow’s regeneration or the migration of unirradiated BM hematopoietic stem cells.^5^ In our center, most of patients receive whole pelvic RT (AP/PA fields) instead of four-field or box technique due to the prior clinical observation. The patients who receive four-field or box RT have a high risk of osteonecrosis of the femoral head and low-control of paraaortic lymph node tumor metastasis.

PCR, as one of the important multivariate calibration methods, can fully utilize all the dosimetric data and repeatedly perform factor analysis to identify the number of components in a mixed sample and predict the components in unknown samples with higher precision than univariate calibration.^16^ PCR offers several analytical advantages and can determine PCs that describe relevant information. Most of the data from PCs contain noise. PCA is a technique used to reduce the dimensionality of a dataset by orders of magnitude to retain the maximum relevant information and to generate a few PCs to replace an entire dataset. This method is different from prior NTCP calculation which can better usage of volume data.

The metrics of the dose volume effect associated with toxicity is based simply on the differences among the parameter values, which are therefore correlated to one another; thus this correlation should be of concern. The complications of multi-collinearity are widely acknowledged but are not an area of focus in this study. More focused research on the strength of this correlation is warranted because it presents a problem for the current methodology. Further studies should be designed with consideration of collinearity. This analytical technique can be applicable to other diseases, such as rectal and anal cancer.

Our report notes that increased numbers of chemotherapy cycles increase the risk of a higher grade of HT. At the same time, we found that the patients with persistent HT were more likely to suffer abnormal blood counts at an early stage of RT and develop severe BM toxicity. Clinicians may therefore wish to be more attentive to patients who have several abnormal blood examination results because these patients have a higher likelihood of break in treatment and missed chemotherapy cycles. All of the patients with HT received drug therapy; however, patients with persistent HT do not have robust blood cells levels and require individually customized treatment strategies.
